# Surgery in Ovarian Cancer—Brazilian Society of Surgical Oncology Consensus

**DOI:** 10.1002/jso.70055

**Published:** 2025-07-29

**Authors:** Audrey Tieko Tsunoda, Ricardo dos Reis, Renato Moretti‐Marques, Glauco Baiocchi, Leonardo Martins Campbell, Angelica Nogueira Rodrigues, Fabio Fin, Carlos Eduardo Mattos da Cunha Andrade, Bruno Roberto Braga Azevedo, Rosilene Jara Reis, Thales Paulo Batista, Reitan Ribeiro, Deraldo Falcao, Gustavo Guitmann, Suzana Arenhart Pessini, Paulo Henrique Zanvettor, João Soares Nunes, José Clemente Linhares, José Augusto Belotti, Alexandre Oliveira, Rodrigo Nascimento Pinheiro

**Affiliations:** ^1^ PPGTS Pontifícia Universidade Católica do Paraná/PUCPR Curitiba Paraná Brazil; ^2^ Robotic Surgery Program and Peritoneal Diseases Center Hospital do Coração (HCor) São Paulo São Paulo Brazil; ^3^ Department of Gynecologic Oncology Hospital Erasto Gaertner Curitiba Paraná Brazil; ^4^ Instituto de Oncologia do Paraná Curitiba Paraná Brazil; ^5^ Department of Gynecologic Oncology Barretos Cancer Hospital Barretos São Paulo Brazil; ^6^ HPV Research Group Barretos Cancer Hospital Barretos São Paulo Brazil; ^7^ Department of Gynecologic Oncology Hospital Israelita Albert Einstein São Paulo São Paulo Brazil; ^8^ Department of Gynecologic Oncology A.C. Camargo Cancer Center São Paulo São Paulo Brazil; ^9^ Santa Lúcia Cancer Center Brasília Distrito Federal Brazil; ^10^ Department of General Medicine Federal University of Minas Gerais (UFMG) Belo Horizonte Minas Gerais Brazil; ^11^ Department of Gynecologic Oncology Hospital São Vicente Curitiba Paraná Brazil; ^12^ Instituto Femina Curitiba Paraná Brazil; ^13^ Barretos School of Health Sciences Dr. Paulo Prata (FACISB) Barretos São Paulo Brazil; ^14^ Hospital São Vicente Curitiba Paraná Brazil; ^15^ Eco Oncologia/CIONC Curitiba Paraná Brazil; ^16^ Hospital Ernesto Dornelles Porto Alegre Rio Grande do Sul Brazil; ^17^ Hospital Mãe de Deus Porto Alegre Rio Grande do Sul Brazil; ^18^ Brazilian Lutheran University (ULBRA) Porto Alegre Rio Grande do Sul Brazil; ^19^ Department of Surgery Instituto de Medicina Integral Professor Fernando Figueira (IMIP) Recife Pernambuco Brazil; ^20^ McGill University Health Center Salvador Quebec Canada; ^21^ Department of Gynecologic Oncology Hospital da Mulher Maria Luiza Costa dos Santos Salvador Bahia Brazil; ^22^ Brazilian National Cancer Institute (INCA) Rio de Janeiro Rio de Janeiro Brazil; ^23^ Americas Hospital Rio de Janeiro Rio de Janeiro Brazil; ^24^ Department of Gynecologic Oncology Federal University of Health Sciences of Porto Alegre (UFCSPA) Porto Alegre Rio Grande do Sul Brazil; ^25^ Department of Gynecologic Oncology Aristides Maltez Hospital Salvador Bahia Brazil; ^26^ AMO Clinic Salvador Bahia Brazil; ^27^ Hospital Erasto Gaertner Curitiba Paraná Brazil; ^28^ Federal University of the State of Rio de Janeiro (UNIRIO) Rio de Janeiro Rio de Janeiro Brazil; ^29^ Federal University of Juiz de Fora (UFJF) Juiz de Fora Minas Gerais Brazil; ^30^ Brazilian Society of Surgical Oncology (SBCO) Copacabana Rio de Janeiro Brazil; ^31^ Brazilian College of Surgeons (CBC) Botafogo Rio de Janeiro Brazil; ^32^ Base Hospital of the Federal District Brasília Distrito Federal Brazil

**Keywords:** Brazilian Society of Surgical Oncology, consensus, epithelial ovarian cancer, surgery

## Abstract

Surgical management in epithelial ovarian cancer (EOC) has a significant impact on overall survival (OS) and progression‐free survival (PFS). The Brazilian Society of Surgical Oncology (BSSO) supported an expert‐led task force for consensus: the best EOC surgery is provided by experienced and specialized trained surgeons in cancer centers. Laparoscopic or radiological staging can predict the possibility of complete cytoreduction (CC0) and help to reduce unnecessary laparotomies. Carcinomatosis and nodal extension should be evaluated at imaging. Multidisciplinary input is essential for determining the need for the selection of patients for surgery and adjuvant chemotherapy in patients with EOC. The BSSO proposes quality assurance criteria and the need for national consensus. Genetic counseling was deemed mandatory for all patients with EOC. This consensus states the final recommendations from BSSO for the management of EOC.

## 1 Introduction

Ovarian cancer is associated with 40%–50% mortality as it is frequently diagnosed at advanced stages. Optimal surgical management is a predictive marker for the overall survival (OS) and progression‐free survival (PFS) in epithelial ovarian cancer (EOC). Complete cytoreduction (R0 or CC0) may involve multiorgan resections and additional complex surgical procedures like peritonectomy and diaphragmatic resections. This is best achieved by a multidisciplinary team of trained and experienced surgeons in cancer care centers. Therefore, centralization of cancer care, where evidence‐based approaches are offered, has become an important demand over the years [1, 2]. New technology and human resources should be distributed according to the surgery′s complexity levels [3]. In the absence of a formal surgical specialty focused on gynecologic cancer treatment, scientific task forces may play an important educational role. The Brazilian Society of Surgical Oncology (BSSO) created a task force on ovarian cancer, initially aiming at establishing minimum standards and a management protocol for surgical treatment and over 200 Brazilian medical professionals have been collaborating toward a digital network for better cancer care. After 8 years of activities, including consensus statements, tumor boards, interdisciplinary educational programs, BSSO proposed an update for this surgical guideline, in order to offer stratified recommendations for the diverse national settings.

## 2 Methods

This initiative united experts representing major surgical and oncological Brazilian societies, from different Brazilian regions and institutions, over the past 8 years. In 2017, over 10 months, 17 leading experts (16 surgeons included) in the management of EOC from 12 cancer centers attended meetings, digital discussions, and performed electronic online paper writing. For this updated version, 19 specialists reviewed the original work and updated all the data according to the current evidence. A comprehensive literature review of the studies published between January 2005 and September 2024 was carried out, and the recommendations were updated. All recommendations were voted for concordance and approval by all members on an electronic platform. This open and collaborative approach allowed all members to participate and express criticism, in addition to a scientific review adjusted to current Brazilian standards. Some questions were answered as consensus statements regarding key issues in EOC management. All recommendations were evaluated according to National Comprehensive Cancer Network guidelines (NCCN) categories of evidence and consensus (Table 1) [4].

**Table 1:** NCCN categories of evidence and consensus.

Category 1	Based upon high‐level evidence, there is uniform NCCN consensus that the intervention is appropriate.
Category 2A	Based upon lower‐level evidence, there is uniform NCCN consensus that the intervention is appropriate.
Category 2B	Based upon lower‐level evidence, there is NCCN consensus that the intervention is appropriate.
Category 3	Based upon any level of evidence, there is major NCCN disagreement that the intervention is appropriate.

*Note:* All recommendations are category 2A unless otherwise indicated.

### 2.1 How Is the Current Situation of Ovarian Cancer?

According to GLOBOCAN, 324 398 new ovarian cancer cases were detected in 2022, and 206 839 women died from the disease [5]. It is the eighth most common cancer and the eighth cause of death from cancer in women [6]. EOC comprises around 95% of malignant ovarian neoplasms [7]. In Brazil, according to the Ministry of Health, 7310 new ovarian cancer cases are expected in 2024, and around 3900 deaths due to the disease yearly. It is the seventh most common cancer among women in the South, North, and Northeast regions [8]. In Brazil, there is incomplete reporting regarding stages at diagnosis, modalities of treatment, access to medication, and surgical standards. São Paulo State organized a compilation of oncological data provided by all state referral cancer centers and some volunteer institutions. Ovarian cancer cases correspond to 3.2% of total cancer diagnoses in women, excluding skin cancer. The mean time for diagnosis in oncologic cases is 14 days, and for primary treatment ranges from 12 days (after diagnosis in cancer centers) to 70 days (diagnosis outside cancer centers) [8].

Since 2012, all Brazilian citizens have a legal right to start a first cancer treatment within 60 days from the diagnosis [9]. Many advanced‐stage ovarian cancer patients could lose performance status during this period and consequently impair their prognosis due to limited treatment options. Centralization of ovarian cancer cases in tertiary cancer centers provides better time intervals, as demonstrated in São Paulo state data, and is completely supported by this panel.

### 2.2 Which Are the Institutions and Who Are the Surgeons That Should Provide Surgical Management for Ovarian Cancer Patients?

*Consensus: Patients with a high risk of an established diagnosis of EOC should be evaluated by a specialized gynecologic oncologist or a surgical oncologist focused on gynecologic cancer treatment. First, surgery should be performed by a high‐volume specialist (undertaking more than 10 procedures per year), preferably in a high‐volume hospital (20 or more cases per year). (Category 2)*

Ovarian cancer mortality depends on a multidisciplinary surgical team operating in a tertiary hospital or a cancer center. Ovarian cancer patients treated by gynecologic oncologists have superior outcomes than those treated by general gynecologists and general surgeons [10].

Guideline‐adherent cancer care is associated with high‐volume hospitals (20 or more cases per year; 50.8% compared with 34.1%; *p *< 0.001) and high‐volume physicians (10 or more cases per year; 47.6% compared with 34.5%; *p *< 0.001) [11, 12]. Adherence to NCCN guidelines for the treatment of ovarian cancer correlates with improved survival and may serve as a useful process measure of quality cancer care [13, 14]. According to the European Society of Gynaecological Oncology (ESGO), quality assurance for referral centers and institutional accreditation may play a significant role to elevate the quality of care in Europe and may be replicable worldwide [15].

### 2.3 Which Are the Best Imaging Tools Prior to Surgical Management?

*Consensus: All patients should have a chest CT scan, and a complete abdominal imaging acquisition, CT or MRI. (Category 2) Relative contraindications to surgery should be discussed in a multidisciplinary tumor board meeting and preoperative surgical planning. (Category 2)*

Imaging methods are useful for diagnosis and to assess the extent of disease. Diffusion magnetic resonance imaging (MRI) is the best preoperative alternative to evaluate peritoneal disease compared to ultrasound (USG), computed tomography (CT), or positron emission tomography (PET)‐CT. Diffusion MRI is more accurate in detecting the presence of disseminated disease, especially for intraperitoneal assessment, hepatic and splenic metastasis, and predicting resectability [16]. Radiologic contraindications to primary debulking surgery are rare and include massive mesenteric infiltration and retraction with segmental small bowel sub occlusion, massive porta hepatis or hepatic round ligament infiltration with elevation of serum bilirubin, liver or lung parenchymal metastases requiring more than three liver segmental resections or more than a lobectomy, omental cake with clear infiltration of the lesser gastric curvature (that would demand total gastrectomy with en bloc transverse colectomy) [17].

### 2.4 Which Are the Best Tools for Selecting Patients for Surgery?

*Consensus: The selection of patients for primary debulking surgery (PDS) or neoadjuvant chemotherapy (NACT) followed by interval debulking surgery (IDS) should be guided by a combination of performance status, age, and tumor burden. (Category 2) Laparoscopic evaluation of tumor spread and resectability is recommended as an effective tool to avoid unnecessary laparotomies, preventing the morbidity associated to nontherapeutic procedures. (Category 2)*

An adequate patient selection for ovarian cancer surgical treatment requires individualized planning that takes into account the individual functional evaluation and disease extension. Historically, the Karnofsky Performance Status (KPS) [18] and Eastern Cooperative Oncology Group (ECOG) [19] scales have been instrumental in accessing patients functional capacity with a significant correlation to oncologic outcomes [20]. More recent studies emphasize that frailty indices, which take into account a broader functional and physical condition, are better predictors of perioperative morbidity and mortality, the ability to complete primary therapy, OS, and PFS than age alone [21].

In addition, nutritional, psychological, and anesthetic risks, as well as comorbidities, should be addressed as they may impact the results of a major surgical procedure. A multidisciplinary evaluation is paramount, especially when a multivisceral resection is planned to achieve a complete resection (R0) [22]. Adequate abdominal peritoneal spread evaluation may predict resectability and surgical complexity. In 2005, Harmon and colleagues standardized a peritoneal cancer index (PCI) in 13 different abdominal regions, each of them classified as 0 (no tumor), 1 (tumors up to 0.5 cm), 2 (tumors up to 5 cm), and 3 (tumor with more than 5 cm in the largest diameter) [23]. Traditionally, this index is calculated independently of the primary tumor during an exploratory laparotomy for carcinomatosis. A PCI score > 20 suggests the need for NACT due to the increased likelihood of surgical complexity and suboptimal debulking with primary surgery.

A laparoscopic score was designed and validated by Fagotti and colleagues in 2006 for ovarian cancer carcinomatosis evaluation [24, 25], and updated in 2015 [26]. It is a binary system targeting resectability criteria, in which each of the following laparoscopic parameters scores 2 points when present: (1) massive peritoneal involvement and a miliary pattern of distribution for parietal peritoneal carcinomatosis; (2) widespread infiltrating carcinomatosis, and confluent nodules to the most of the diaphragmatic surface; (3) tumor diffusion along the omentum up to the large stomach curvature; (4) possible large/small bowel resection (excluding, rectosigmoid involvement, giving its pelvic localization and since posterior exenteration is considered a standard surgical procedure in Advanced Epithelial Ovarian Cancer [AEOC]); (5) evident neoplastic involvement of the stomach, and/or lesser omentum, and spleen; and (6) liver surface lesions larger than 2 cm. Since mesenteric retraction and miliary carcinomatosis on the small bowel serosa are widely recognized as absolute criteria of unresectability, these criteria were excluded from the score [26].

The higher the score, it is less likely that the patient will be optimally debulked at surgery. In the updated version of the score, patients with 10 or more points had no chance of complete cytoreduction with primary surgery. On the other hand, with a score of 8, only 8.3% of the patients achieve complete cytoreduction [26].

The decision‐making process to perform extensive surgical procedures must take on:

– Extensive preoperative planning;

→ If carcinomatosis is so spread that it does not allow a primary complete cytoreduction, this has to be assessed by a trained surgical team.

– Multidisciplinary meeting;
– Careful analysis if there are clinical or anesthetic contraindications for primary surgery.

### 2.5 What Are the Main Surgical Objectives?

*Consensus: Complete R0 resection is the primary goal, with acceptable morbidity. (Category 1) Primary debulking surgery is the mainstay approach. (Category 1) Patients without good surgical conditions or/and excessive tumor burden should be considered for neoadjuvant chemotherapy and interval debulking surgery. (Category 2)*

Surgical cytoreduction in the treatment of EOC has been the object of study since Griffiths in 1975 [27], which related more remarkable survival with smaller residual lesion size. Blythe and Wahl in 1982 [28] demonstrated more remarkable survival in patients with residual lesions up to 2 cm. Piver in 1986 [29] described the possibility of reaching this pattern of cytoreduction in 75% of stages III and IV. The GOG study published by Hoskins et al. in 1994 [30] concluded that residual disease up to 2 cm was related to better OS of the patient. In 2007, the German Group (AGO‐OVAR) [31] published a prospective randomized trial demonstrating that residual disease is an independent prognostic, and patients with no residual disease presented a better prognosis. In 2009, du Bois et al. [32] published data from a combined exploratory analysis of three prospective randomized phase 3 trials. The results confirmed that the primary goal of ovarian cancer cytoreductive surgery should be no visible residual disease.

In this context, the PCI, developed by Sugarbaker, originally applied in the gastrointestinal neoplasms, describes the extent of the disease, and the residual tumor should be defined following the size of the larger tumor diameter of the residual disease (Table 2). Therefore, the adoption of PCI as part of the preoperative evaluation represents not only a legacy of Sugarbaker's systematized approach, but also a consolidated clinical strategy in the search for better oncological outcomes in advanced ovarian cancer [33, 34].

**Table 2:** Sugarbaker's completeness of cytoreduction score (adapted from Sugarbaker [33]).

Completeness of cytoreduction score	Volume of disease at the end of surgical procedure
CC‐0	No visible peritoneal carcinomatosis after CRS
CC‐1	Nodules persisting < 2.5 mm after CRS
CC‐2	Nodules persisting between 2.5 mm and 2.5 cm
CC‐3	Nodules persisting > 2.5 cm

Abbreviation: CRS, cytoreductive surgery.

Two major randomized trials proved that patients with stages IIIC–IV EOC and primary upfront surgery achieved comparable oncological outcomes to interval debulking surgery patients [35, 36]. Most patients from both studies were ECOG PS 0 or 1. Upfront surgery was related to more multiorganic resections and morbidity. In both studies, R0 rates in both arms were comparable to non‐specialists performing surgery in general practice centers (18.3% and 16% PDS vs. 48% and 43% NACT, respectively). The median OS was 29 versus 24.1 months for PDS and 30 versus 22.6 months for NACT [35, 36]. The morbidity of an extensive cytoreductive surgery varies from 30% to 60% and should be balanced, with a target of starting adjuvant chemotherapy within 4–6 weeks after an operation.

Other clinical trials that evaluated the same approach were the Onda and SCORPION trials. The Onda trial (JCOG0602) was a phase III clinical trial, in stages III/IV ovarian, fallopian tube, or primary peritoneal cancer, that confirmed the noninferiority of NACT, suggesting that NACT may not always be an adequate substitute for PDS [37]. The SCORPION trial, a phase III randomized clinical trial, showed that the NACT group had lower rates of severe postoperative complications and higher rates of complete resection. However, there was no significant difference in PFS or OS [38].

Despite complex and extensive surgical approaches that may enhance rates of completeness of cytoreduction, tumor load remains an independent poor prognostic factor despite and probably reflects a more aggressive biological behavior of the tumor [39, 40]. This highlights the need for a comprehensive multimodal approach to improve outcomes in EOC.

The KELIM score (Kinetic Elimination of CA‐125) is a prognostic biomarker based on the kinetics of the tumor marker CA‐125 during the first 100 days of chemotherapy in patients with EOC. It quantifies the tumor's intrinsic chemosensitivity by analyzing the rate at which CA‐125 declines, offering a predictive insight into treatment response and long‐term outcomes. A favorable KELIM score (high elimination rate) is associated with better OS and PFS, while an unfavorable KELIM score (low elimination rate) suggests resistance to platinum‐based chemotherapy [41, 42].

### 2.6 What Is the Role of Chemotherapy in EOC, and How Does It Fit Surgical Treatment?

*Consensus: Chemotherapy is necessary for most EOC patients. Platinum‐based regimens are preferred as a first‐line therapy, and for platinum‐sensitive recurrent ovarian cancer (Category 1), Intraperitoneal (IP) chemotherapy can be offered for selected patients after complete primary cytoreduction. (Category 1)*

Patients with initial EOC stage I and grade 3, or stage IC should be completely staged, and may benefit from only three cycles of platinum‐based chemotherapy [43]. When considering a serous histology tumor, there is a potential benefit from six cycles in this early‐stage high‐risk group of patients [44]. For women with completely resected grade 1, stages IA or IB EOC, observation alone is suggested.

Primary debulking surgery followed by platinum‐based systemic chemotherapy is the initial recommendation for EOC medically fit patients, stages II–IV. Adjuvant therapy is platinum‐based and is less morbid when carboplatin is associated to paclitaxel, for six cycles, on average [45]. The combination of pegylated liposomal doxorubicin to carboplatin was not superior to a standard‐of‐care carbo‐paclitaxel regimen [46].

Patients with bulky stages III–IV disease, poor surgical candidates and/or with tumor load unlikely to be optimally debulked, assessed by a specialist gynecologic oncologist, are potential candidates for interval debulking surgery. Morbidity is reduced in this approach, with a comparable DFS and OS [35, 36].

Hyperthermic perioperative chemotherapy (HIPEC) with cisplatin (100 mg/m^2^) can be offered for interval cytoreductive patients completely or optimally debulked. In a recent RCT intention‐to‐treat analysis, morbidity was comparable and a significant 3.5 months median DFS improvement was achieved in the HIPEC group (14.2 vs. 10.7 months) [47]. HIPEC seems to be a promising tool in ovarian cancer therapy, and its use is currently the objective of prospective trials. It can be an option for medically fit patients, treated in centers of excellence, by trained surgeons, after a multidisciplinary tumor board assessment, and at least stable disease after neoadjuvant chemotherapy (Category 3).

Evaluation of BRCA mutation status and homologous recombination deficiency (HRD) status at diagnosis is the ideal approach for all patients diagnosed with high‐grade nonmucinous epithelial carcinoma, for planning therapeutic management and hereditary cancer prevention strategies. Maintenance treatment in patients with stages III and IV high‐grade serous and endometrioid subtypes with BRCA mutation should preferably include olaparib or niraparib. Olaparib and bevacizumab can be used in selected cases. Maintenance treatment in patients with stages III and IV high‐grade serous and endometrioid subtypes without BRCA mutation and HRD positive should include olaparib and bevacizumab or niraparib. Maintenance treatment in patients with stages III and IV high‐grade serous and endometrioid subtypes without BRCA mutation and HRD negative can consider the use of niraparib or bevacizumab [48, 49, 50, 51, 52, 53, 54, 55, 56].

### 2.7 Which Are the Main Quality Characteristics to be Assessed?

*Consensus: Quality assessment is paramount to achieve better outcomes. Primary cytoreductive surgery (> 50%), procedures performed by specialists (> 90%), rate of R0 resections (> 65%), completeness of surgical, pathological and morbidity records, operative structure, multidisciplinary treatment planning are the recommended performance indicators for quality assurance in ovarian cancer surgery. (Category 2)*

Quality of care is defined as “the degree to which health services for individuals and populations increase the likelihood of desired health outcomes and are consistent with current professional knowledge.” Good quality means providing patients with appropriate services in a technically competent manner, with good communication, shared decision‐making, and cultural sensitivity. Quality assurance can be defined as all those planned and systematic actions necessary to provide adequate confidence that a product or service will satisfy given requirements for quality [57, 58].

In ovarian cancer, the quality of the surgical procedures (staging and cytoreductive surgeries) is the cornerstone of patient care. Although, solid data have shown that patients treated by gynecologic oncologists and in specialized centers have better outcomes, heterogenicity in surgical care still exists [59, 60, 61]. The identification of surgical quality indicators is challenging due to the lack of qualitative parameters [62].

Two international expert agreements were published after the identification of process‐based quality indicators for early and advanced‐stage ovarian cancer. In 2009, the European Organization for Research and Treatment of Cancer (EORTC) [63] proposed quality indicators for staging laparotomy in EOC grossly confined to the pelvis and for primary debulking surgery for stages III–IV EOC. Yet, the European Society of Gynecologic Oncology (ESGO) [15] also proposed in 2016 a complete list that included 10 QIs for advanced EOC surgery that can be used to audit and improve the clinical practice (Table 3) [64, 65, 66].

**Table 3:** Advanced (stages III–IV) ovarian cancer surgery quality indicators, modified from ESGO [15, 64].

Indicator	Target	Observation
Rate of complete surgical resection	– Optimal target: ≥ 65%	Complete abdominal surgical resection is defined by the absence of remaining macroscopic lesions after careful exploration of the abdomen.
	– Minimum required target ≥ 50%	
	– Proportion of primary debulking surgeries: ≥ 50%	
Number of cytoreductive surgeries performed per center and per surgeon per year	Number of surgeries performed per center per year:	Only surgeries with an initial objective of complete cytoreduction are recorded.
		≥ 95% of surgeries are performed or supervised by surgeons operating at least 10 patients a year.
	– Optimal target: *N* ≥ 100	
	– Intermediate target: *N* ≥ 50	
	– Minimum required target: *N* ≥ 20	
	– Minimum required target: *N* ≥ 20	
Surgery performed by a gynecologic oncologist or trained surgeon specifically dedicated to gynecological cancer management	≥ 90%	Surgery is performed by a certified gynecologic oncologist or, in countries where certification is not organized, by a trained surgeon dedicated to the management of gynecologic cancer (accounting for over 50% of his practice) or having completed an accredited fellowship.
Center participating in clinical trials in gynecologic oncology	Yes	The center actively accrues patients in clinical trials in gynecologic oncology.
Treatment planned and reviewed at a multidisciplinary team meeting	≥ 95%	The decision for any major therapeutic intervention has been taken by a multidisciplinary team (MDT) including at least a surgical specialist, a radiologist, a pathologist (if a biopsy is available), and a physician certified to deliver chemotherapy.
Required preoperative workup	≥ 95%	Unresectable parenchymal metastases have been ruled out by imaging. Ovarian and peritoneal malignancy secondary to gastrointestinal cancer has been ruled out by suitable methods, e.g., plasma CA125 and CEA levels, and/or by biopsy under radiologic or laparoscopic guidance.
Pre‐, intra‐, and postoperatory management	Not applicable	The minimal requirements are: (i) an intermediate care facility, and access to an intensive care unit in the center are available, (ii) an active perioperative management program is established.
		Follow the ERAS (Enhanced Recovery After Surgery) protocol, which includes practices such as preoperative nutrition, multimodal analgesia, early mobilization, and fluid volume optimization [65].
Minimum required elements in operative reports	90%	Operative report is structured. Size and location of disease at the beginning of the operation must be described. All the areas of the abdominal cavity must be described. If applicable, the size and location of residual disease at the end of the operation, and the reasons for not achieving complete cytoreduction must be reported.
Certification	Centers with ≥ 20 cases/year and a score ≥ 32/40 are eligible for certification.	Centers of Excellence: ≥ 50 cases/year, PI of a clinical trial, peer‐reviewed publication in the last 3 years, and ESGO fellowship accreditation.
		ESGO Certification 2020 (updated) [64].
Minimum required elements in pathology reports	≥ 90%. The tolerance within this target reflects situations where it is not possible to report all components of the data set due to poor quality of specimen.	Numerator: number of patients with advanced ovarian cancer undergoing cytoreductive surgery who have a complete pathology report that contains all cytoreductive surgery who have a complete pathology report that contains all.
		ICCR (International Collaboration on Cancer Reporting) must comply with the latest ICCR standards 2022: standardize pathology reports, ensuring consistency and quality in communication between pathologists and clinical teams, as well as facilitating data collection for oncology research and registries [66]
Existence of a structured prospective reporting of postoperative complications	– Optimal target: 100% of complications are prospectively recorded	Data to be recorded are reoperations, interventional radiology, readmissions, secondary transfers to intermediate or intensive care units, and deaths
	– Minimum required target: selected cases are discussed at morbidity and mortality	

### 2.8 Surgical Major Technical Issues

*Consensus: Advanced surgical skills provided by surgical specialists, in a referral center with a multidisciplinary approach, are the best combination to reduce morbidity and enhance the surgical objectives of cytoreduction. (Category 2)*

*Surgeons must provide a detailed surgical report describing the extent of initial disease before debulking pelvis, mid‐abdomen, and/or upper abdomen. Also, the amount of residual disease in the same areas after debulking. Complete or incomplete resection, and if incomplete, indicate the size of the major lesion and total number of lesions. (Category 2)*

Surgical aims in ovarian cancer include diagnosis for adnexal mass or carcinomatosis, accurate staging for limited disease, peritoneal extension assessment, or cytoreduction for advanced or metastatic disease.

#### 2.8.1 Patient and Anesthesia Requirements

Any institution interested in performing ovarian cancer surgery must have an intermediate high dependency care facility and access to an intensive care unit in the center. An active perioperative management program is important and should include preoperative nutritional and hemoglobin optimization, with iron deficit correction. According to the current guidelines, fluid management (Goal Directed Therapy) and pain management should include an option of epidural analgesia to reduce opioid demand. Routine premedication is no longer recommended. Preemptive nausea and vomiting medications should be systematic [15].

#### 2.8.2 Surgical Initial Technical Aspects

After a multidisciplinary meeting and considering the disease as potentially resectable in the preoperative imaging review, a staging laparoscopy is advisable. All patients with PS2 (ECOG Performance Status 2) or more, but fit for general anesthesia, should be scheduled for laparoscopy first. This may provide more detailed information regarding the extension of disease, diagnosis, and documentation for neoadjuvant chemotherapy and further response assessment. Patients with good PS, but high tumor load, should also be scheduled for staging laparoscopy. After the procedure, the multidisciplinary team may provide further details regarding organs to be spared or resected, and morbidity and mortality in upfront versus interval debulking procedures.

Patients with a combination of good PS and potentially resectable disease, with few or no organs to be resected, can be scheduled for staging laparoscopy followed by cytoreductive procedure, under a single anesthetic procedure. This patient selection potentially reduces unnecessary complex anesthetic procedures for patients that are not suitable for a primary cytoreduction and improves the operating rooms timetable. Although a two surgical step approach is not comfortable for the patient (two different admissions and anesthetic procedures), it has the potential to spare significant time and improve OR optimization. Staging laparoscopy (S‐LPS), also known as the Fagotti score [24], is the most feasible score for selecting patients with EOC to upfront surgery or neoadjuvant treatment. It consists of minimally invasive surgery (MIS) staging prior to a definitive cytoreductive attempt. For this reason, surgery should start with an umbilical incision for laparoscopic inspection. A second port can be inserted in the lower abdomen to help with bowel manipulation, preferably on the middle line. In case of advanced disease, an extra 5‐mm port should be inserted in the midline in the upper abdomen, and the Fagotti score applied laparoscopic approaches in patients with carcinomatosis without a complete cytoreductive surgery at the same time may result in a high rate of trocar implants. Those sites should have to be resected after responding to chemotherapy, and that is the main reason why it is not recommended to insert ports outside the middle line when a complete cytoreductive procedure is not intended on the same surgical procedure [67, 68, 69].

#### 2.8.3 Role of Surgical Staging

For initial EOC, stages I and II, the accuracy and adequacy of surgical staging by laparotomy or MIS appear to be equivalent, with neither approach conferring a survival advantage. Minimally invasive approaches result in lower postoperative complication rates, shorter postoperative hospital stay [70], and less blood loss [71]. However, intraoperative tumor rupture has been reported to occur more frequently in patients undergoing laparoscopy compared with laparotomy in retrospective cohort studies, mainly during the MIS learning curve [72]. Until this moment, there is no randomized data comparing laparotomy and laparoscopy staging for ovarian cancer [73], and probably it will never be performed, due to the paucity of cases.

#### 2.8.4 Surgical Technical Details

A surgical staging starts with aspiration of ascites or peritoneal wash for peritoneal cytologic examination. Complete peritoneal surface evaluation, followed by excision of any peritoneal surface or adhesion suspicious for metastasis, should be performed. In the absence of any suspicious areas, random peritoneal biopsies should be taken from the pelvis, paracolic gutters, and both diaphragmatic surfaces.

Bilateral salpingo‐oophorectomy and total hysterectomy should be performed with every effort to keep an encapsulated mass intact during removal. For selected patients desiring to preserve fertility, uterine and contralateral adnexal preservation may be considered if these structures are not involved by tumor. Infracolic omentectomy should be performed [74].

Retroperitoneal lymphadenectomy includes the removal of nodal tissue from the vena cava and the aorta bilaterally, up the left renal vein. Systematic pelvic lymphadenectomy contemplates nodal tissue from the common, internal and external iliac vessels and obturator fossa superficial to the obturator nerve. For advanced stage disease (IIB–IV), and in the absence of macroscopic and preoperative imaging of suspicious lymph nodes, after complete cytoreductive surgery, systematic lymphadenectomy increased morbidity without OS benefit, in a recent trial presented in an oncological meeting [75].

Also, the CARACO abstract trial presented at ASCO 2024 showed that adding retroperitoneal lymphadenectomy to complete cytoreductive surgery, in primary surgery or in interval surgery after neoadjuvant chemotherapy in patients treated for an advanced ovarian cancer, with no suspicious nodes, does not improve PFS nor OS. It is important to highlight that 75% of the patients were treated with neoadjuvant chemotherapy, most of them after 3–4 cycles [76].

The goal of cytoreductive surgery in ovarian cancer should be a complete resection to no visible residual disease [32]. Therefore, advanced techniques of peritoneal stripping, multivisceral resection, and upper abdominal management [77, 78] have been standardized and combined to surgical staging techniques (Table 4).

**Table 4:** Epithelial ovarian cancer surgical treatment according to clinical stages.

Stages IA or IC fertility preserving desire	Unilateral salpingo‐oophorectomy and comprehensive surgical staging^c^
Stage IB fertility preserving desire	Bilateral salpingo‐oophorectomy and comprehensive surgical staging^c^
Stages I–IV good performance status and mild^a^ to moderate tumor load, no fertility preserving desire	Total hysterectomy with bilateral salpingo‐oophorectomy and comprehensive surgical staging^c^ and debulking with complete (or optimal) cytoreduction as a surgical goal
Stages III–IV high tumor load and/or poor surgical candidate^b^	Neoadjuvant chemotherapy followed by surgical reassessment for interval debulking surgery

^a^ Adnexal mass with limited disease and no fertility desire could benefit from frozen section and proceed as indicated approach.

^b^ Poor surgical candidates may benefit from percutaneous biopsy with histologic diagnosis or a combination of diagnostic cytology and CA125/CEA ratio > 25.

^c^ Laparoscopic or robotic approach acceptable and advisable, preserving oncological principles of avoiding tumor spillage and performance of a comprehensive peritoneal cavity inspection.

### 2.9 Peritonectomy Technique

#### 2.9.1 Greater/Infracolic Omentectomy

The greater omentum is elevated off the transverse mesocolon by stripping the entire surface of the mesocolon. The dissection includes separation of the specimen from the gastroepiploic vessels (preserved if possible) and division of the short gastric vessels. The omentum is dissected and detached from the splenic hilum and the anterior surface of the pancreas. Meticulous dissection of the omentum is essential for complete tumor removal.

#### 2.9.2 Epigastric Peritonectomy

The falciform ligament is separated from the umbilicus along with the anterior peritoneum and resected flush with the liver surface to include the ligamentum teres hepatis. A bridge of liver may be divided to access the left portal vein if necessary.

#### 2.9.3 Right Hemidiaphragmatic Peritonectomy

Diaphragmatic peritoneum is stripped along its entirety after making a cruciate incision in the anterior peritoneum. The peritonectomy may include stripping the Gerota fascia, the right adrenal gland surface, and the liver Glisson capsule. A ventral liver mobilization technique for the cytoreduction of diaphragmatic tumors with involvement of the liver is feasible and safe. Recognition of upper abdominal anatomy and liver mobilization maneuvers is fundamental to allow exploration and debulking of the diaphragm, reducing the risk of major vessel injuries (retrohepatic caval vein, hepatic hilum, supra‐hepatic veins, diaphragmatic vessels) [78]. The specific sequence of liver mobilization varies from patient to patient according to the tumor distribution and extension [78, 79]. The retrohepatic inferior vena cava (IVC) is referred to as the medial border of the dissection. This surgical procedure can be adopted for the management of bulky diaphragmatic tumors in select patients [80].

#### 2.9.4 Left Hemidiaphragmatic Peritonectomy

The upper left portion of a cruciate incision is used to initiate the left hemidiaphragmatic peritonectomy. Complete stripping of the diaphragmatic fibers with skeletonization (or ligation) of the inferior phrenic vessels may be undertaken. Dissection may include stripping the adrenal gland surface and Gerota fascia.

#### 2.9.5 Lesser Omentum Resection

The hepatoduodenal ligament and the pars flaccida are dissected from the caudate lobe of the liver and the porta hepatis. Careful dissection of the celiac axis branches and the right gastric arteries can elevate the tumor off the lesser omentum. The IVC bursa is occasionally stripped, using the IVC, caudate lobe of the liver, and the left limb of the right crus as anatomical landmarks.

#### 2.9.6 Pelvic Peritonectomy

Pelvic peritonectomy includes resection of the anterior peritoneum with or without the urachus and the medial umbilical ligaments. A rectal and Douglas pouch shaving may reduce morbidity with a complete pelvic peritoneal clearance. Visceral resections of the uterus and ovaries are performed as necessary.

#### 2.9.7 Anterior Peritonectomy

Scar excision and resection of the anterior peritoneum is carefully undertaken with preservation of the rectus muscle fascia as the procedure starts. This becomes contiguous with the other peritonectomy specimens in the presence of extensive peritoneal disease [81].

#### 2.9.8 Video‐Assisted Thoracic Surgery (VATS)

VATS should be considered for incorporation into the standard management algorithm for patients with advanced EOC and pleural effusion. VATS allows assessment of intrathoracic disease and may select candidates for primary cytoreductive surgery and possible intrathoracic cytoreduction versus neoadjuvant chemotherapy [82, 83].

### 2.10 Is Genetic Counseling Mandatory?

*Consensus: Upon deciding the first line systemic treatment, the molecular analysis of all EOC is mandatory, to direct patients with homologous recombination deficiency (HRDd) to parp inhibitor treatment. And with above molecular analysis, performing somatic BRCAm tests has the potential to select the patients to a posterior genetic counseling*.

*There is a formal recommendation for referring all patients with EOC to genetic counseling, regardless of age or familiar history. (Category 2) In the absence of a specialized geneticist, all oncologists should be trained to offer BRCA 1 and 2 testing for all patients with serous epithelial ovarian cancer. (Category 2) Patients with positive criteria for other genetic syndromes or familial clustering should be referred for an oncogeneticist*.

Approximately 24% of all patients with nonmucinous carcinoma of ovarian, peritoneal, or fallopian tube carried germ‐line loss‐of‐function mutations, 18% in BRCA1 or BRCA2. More than 30% of patients have no family history of breast or ovarian carcinoma, and > 35% are 60 years or older at diagnosis [84]. Among EOC Brazilian′s patients unselected for family history of cancer, approximately 19% are BRCA1/2 germline mutation carriers [85].

Based on this information, comprehensive genetic testing for inherited carcinoma is highly recommended for all women with ovarian, peritoneal, or fallopian tube carcinoma, regardless of age or family history. The overall rearrangement frequency is relatively uncommon in the Brazilian population [85, 86], but if NGS or Sanger sequencing do not confirm single‐nucleotide variants (SNVs), or small insertions and deletions (indels), array comparative genomic hybridization (aCGH) or multiplex ligation‐dependent probe amplification (MLPA) should be used for rearrangement diagnosis.

Despite negative BRCA1 and BRCA2 test results, in certain cases, if clinicians remain suspicious of another hereditary cancer syndrome due to the family history of cancer, multigene panel testing should be considered [87]. Risk‐reducing salpingo‐oophorectomy remains the standard of care to reduce ovarian cancer mortality among women at increased risk. Salpingectomy in women with Hereditary Breast and Ovarian Cancer Syndrome should be offered only in the context of a clinical trial [88].

### 2.11 Is There a Role for Surgery in EOC Recurrence?

*Consensus: All patients with recurrence should be referred to multidisciplinary tumor board for case review and best management decision. (Category 2)*

Salvage surgery should be offered for surgically fit patients, after a multidisciplinary tumor board assessment, considering platinum sensitivity, performance status, tumor load, resectability, and absence of ascites [89]. We need to consider the three recently published trials for clinical decision‐making. In GOG 213, there was no difference in PFS and OS when comparing surgery to no surgery. However, in SOC 1, PFS was superior in the surgery group, with no difference in OS. Finally, in DESKTOP III, surgery was superior in both PFS and OS [90, 91, 92].

Platinum‐sensitive patients with first, second, third, and even fourth relapses are potential candidates for surgery and should be referred for a specialized surgical evaluation. A complete secondary cytoreduction in platinum‐sensitive patients significantly improves OS. Thus, the main goal for surgery should be similar to primary debulking surgery: to achieve a complete resection to no macroscopic residual tumor. When not possible, as a minimum procedure, optimal cytoreduction up to 0.5–1 cm residual tumor should be considered [2].

### 2.12 When Is Palliative Surgery Indicated?

*Consensus: Patients with significant symptoms that may be relieved by surgery should be referred to surgical evaluation and tumor board meeting. (Category 2)*

Patients with pain due to a resectable metastatic tumor, bleeding, or a specific segmental bowel occlusion may benefit from palliative surgery [93]. Patients with ovarian cancer may experience higher rates of bowel occlusion. Palliative disobstructive surgery (limited segmental resection and/or stoma) is indicated in surgically fit patients, with a segmental occlusion, without extensive carcinomatosis and, preferably, in the absence of ascites [94].

Incomplete resections are not related to a curative intent, and may negatively impact time to palliative systemic therapy, or even impair quality of life due to surgical complications.

### 2.13 Future Perspectives and Key Questions

Medical societies have been supporting continuous education and national policies for cancer management in Brazil. This consensus supports significant policies for the public healthcare system in the country, including:

– Centralization of ovarian cancer cases in cancer centers.
– Multidisciplinary tumor boards and patient‐driven decisions.
– Quality assurance in ovarian cancer surgery.

Some questions will need to be addressed after this consensus:

– Is laparoscopy the best tool to access resectability in a nationwide perspective?
– Are time intervals acceptable in cancer centers from a heterogeneous large country?
– What are the actual rates of primary debulking surgery and interval debulking surgery?
– What are the oncological outcomes?
– What is the genetic profile of Brazilians treated in the public system?
– What are the main limitations and barriers for adequate treatment?
– What is the role of robotic surgery for staging in early‐stage EOC or in small‐volume disease?

## 3 Conclusions

Surgery has a significant impact on EOC outcomes. This consensus document aims to provide answers to most questions related to the surgical management of EOC patients.

The surgical aim is complete cytoreduction (R0), with acceptable morbidity. This document is a Brazilian guideline and may serve as a tool for governmental decisions regarding new technology and resources acquisition.

## Author Contributions

Audrey Tieko Tsunoda was responsible for conception, planning, carrying out, structuring the group discussions, reviewing the literature, analyzing and writing up, and reviewing this paper. Ricardo dos Reis was responsible for structuring the group discussions, reviewing the literature, analyzing and writing up, and reviewing this paper. Rosilene Jara Reis was responsible for conception, planning, carrying out, structuring the group discussions, reviewing the literature, analyzing and writing up, and reviewing this paper, with the same workload and participation as Audrey Tieko Tsunoda. Reitan Ribeiro, Carlos Eduardo Mattos da Cunha Andrade, Renato Moretti‐Marques, Glauco Baiocchi, Fabio Fin, Angelica Nogueira Rodrigues, Paulo Henrique Zanvettor, Deraldo Falcao, Thales Paulo Batista, Bruno Roberto Braga Azevedo, Gustavo Guitmann, Suzana Arenhart Pessini, João Soares Nunes, Leonardo Martins Campbell, and José Clemente Linhares were responsible for discussing, reviewing the literature, analyzing, partially writing up, and final reviewing this paper.

## Ethics Statement

Due to the scientific review nature of this paper as a specialist consensus, there was no need for ethics committee approval. This paper was approved by the Brazilian Society of Surgical Oncology (BSSO) board of directors and educational members.

## Conflicts of Interest

The authors do not have relevant financial, personal, political, intellectual, or religious interests. Audrey Tieko Tsunoda received honorary for educational lectures and/or advisory board activities for Roche, AstraZeneca, MSD, GSK, and Johnson & Johnson. Renato Moretti‐Marques received honorary for educational lectures for Roche and AstraZeneca. Reitan Ribeiro received honorary for educational lectures and material development, and consulting from Roche, and educational lectures from Johnson & Johnson, GSK, MSD, and AstraZeneca. Carlos Eduardo Mattos da Cunha Andrade received honorary for educational lectures and material development from AstraZeneca, without competing interests with this study.

## Synopsis

This consensus statement by the Brazilian Society of Surgical Oncology (BSSO) provides comprehensive, evidence‐based recommendations for the surgical management of epithelial ovarian cancer (EOC), emphasizing complete cytoreduction (R0) as the primary goal. The document details patient selection criteria, imaging and staging tools, surgical techniques, systemic therapy integration, and institutional quality indicators tailored to the Brazilian healthcare context. It also advocates for centralization of care, multidisciplinary collaboration, and genetic counseling as essential components of high‐quality EOC treatment.

## Data Availability

This article is based on published literature and expert consensus; no new data were created or analyzed in this study. Therefore, data sharing does not apply to this article.
